# The Use of Footstep Sounds as Rhythmic Auditory Stimulation for Gait Rehabilitation in Parkinson’s Disease: A Randomized Controlled Trial

**DOI:** 10.3389/fneur.2018.00348

**Published:** 2018-05-24

**Authors:** Mauro Murgia, Roberta Pili, Federica Corona, Fabrizio Sors, Tiziano A. Agostini, Paolo Bernardis, Carlo Casula, Giovanni Cossu, Marco Guicciardi, Massimiliano Pau

**Affiliations:** ^1^Department of Life Sciences, University of Trieste, Trieste, Italy; ^2^Department of Pedagogy, Psychology, Philosophy, University of Cagliari, Cagliari, Italy; ^3^AOB “G. Brotzu” General Hospital, Cagliari, Italy; ^4^Department of Mechanical, Chemical and Materials Engineering, University of Cagliari, Cagliari, Italy

**Keywords:** rhythm, ecological sounds, auditory stimuli, rhythmic auditory stimulation, Parkinson disease, gait, spatio-temporal parameters, gait analysis

## Abstract

**Background:**

The use of rhythmic auditory stimulation (RAS) has been proven useful in the management of gait disturbances associated with Parkinson’s disease (PD). Typically, the RAS consists of metronome or music-based sounds (artificial RAS), while ecological footstep sounds (ecological RAS) have never been used for rehabilitation programs.

**Objective:**

The aim of this study was to compare the effects of a rehabilitation program integrated either with ecological or with artificial RAS.

**Methods:**

An observer-blind, randomized controlled trial was conducted to investigate the effects of 5 weeks of supervised rehabilitation integrated with RAS. Thirty-eight individuals affected by PD were randomly assigned to one of the two conditions (ecological vs. artificial RAS); thirty-two of them (age 68.2 ± 10.5, Hoehn and Yahr 1.5–3) concluded all phases of the study. Spatio-temporal parameters of gait and clinical variables were assessed before the rehabilitation period, at its end, and after a 3-month follow-up.

**Results:**

Thirty-two participants were analyzed. The results revealed that both groups improved in the majority of biomechanical and clinical measures, independently of the type of sound. Moreover, exploratory analyses for separate groups were conducted, revealing improvements on spatio-temporal parameters only in the ecological RAS group.

**Conclusion:**

Overall, our results suggest that ecological RAS is equally effective compared to artificial RAS. Future studies should further investigate the role of ecological RAS, on the basis of information revealed by our exploratory analyses. Theoretical, methodological, and practical issues concerning the implementation of ecological sounds in the rehabilitation of PD patients are discussed.

**Clinical Trial Registration:**

www.ClinicalTrials.gov, identifier NCT03228888.

## Introduction

Individuals affected by Parkinson’s disease (PD) typically exhibit motor (e.g., tremor, rigidity, postural instability, gait disturbance) and non-motor symptoms, which progressively affect their quality of life. In order to cope with motor symptoms, patients are generally treated with pharmacological therapies (e.g., l-DOPA, dopamine agonists). However, the symptoms tend to become more severe with the progression of the disease and, at the same time, they become more resistant to medication (1), determining the gradual increase of doses and, consequently, the onset of serious side effects. To optimize the use of medication and cope with patients’ impairments, pharmacological therapies are usually accompanied by physical therapy, which is essential for effectively contrasting the motor symptoms and (at least partially) restoring the motor functions. Given that the loss of motor functions increases the risk of falling and gradually affects patients’ independence, researchers have directed their attention to the methods enhancing the efficacy of physical therapy. One of them is the rhythmic auditory stimulation (RAS) developed by Thaut and colleagues (2) and widely studied in the past 20 years (3–6).

The RAS method consists in a gait training, in which patients’ gait is guided by an auditory rhythm. Typically, patients are provided with auditory rhythms (metronome or music), whose beats per minute (BPM) depends on patients’ cadence (steps per minute) at baseline. Usually the BPM is equal to one’s own cadence or slightly increased/decreased (e.g., ±5–10%), depending on the characteristics of the patients and on the methodological choices of the researchers (7–13). The logic of RAS can be understood by analyzing the source of gait disturbance in PD. The damages of basal ganglia, typical of PD, would compromise the functionality of patients’ internal clock, consequently affecting the coordination and the execution of movements (14, 15). Thus, the dysfunctions of the internal clock would be one of the causes of the scarce movement fluidity and of gait impairment. In order to reduce these symptoms, it is necessary to “guide” the internal clock and this can be done by using an external rhythm, that is, RAS. Hence, RAS would facilitate the activity of the internal clock and would help in regulating the fluidity of muscular activation, improving coordination, and facilitating the execution of automatic movements, such as walking. The neural mechanisms underpinning the effectiveness of RAS are not totally clear; it has been proposed that RAS would either rely on residual activity in cortico-striatal circuitry or facilitate compensation by bypassing the damaged areas and relying on alternative pathways (e.g., cerebello-thalamo-cortical circuitry) (16).

The first empirical evidence supporting the efficacy of RAS in the rehabilitation of PD patients was provided by Thaut and colleagues (2). In their study participants were randomly assigned to one of three conditions: RAS, self-paced training, and no training. In both RAS and self-paced training, participants performed daily walking and other exercises for 30 min, for 3 weeks. The only difference was that the RAS group did the exercises with the auditory stimulation, while the self-paced group had no external triggers. The results revealed that both training groups improved in terms of spatio-temporal parameters; however, the RAS group exhibited significantly better results than the other two groups in step cadence, gait speed, and stride length. Significant results were obtained also as concerns the EMG activity of the leg muscles.

In the subsequent years, the efficacy of RAS has been widely confirmed by many studies [for reviews see Ref. (3–6)]. For instance, it has been shown that during a RAS session there is a close synchronization between auditory rhythm and cadence in both PD and healthy participants, suggesting that rhythmic entrainment occurs even with damaged basal ganglia (17). Other studies investigated the immediate effects of RAS (18–29) and its role in training protocols (8, 9, 11–13, 30–32), manipulating important variables (e.g., number of weeks and sessions, duration of each session, tempo of the stimuli), and the majority of them consistently reported positive effects of RAS. The improvements of training protocols have been observed in different kinds of variables: (1) clinical measures, such as unified Parkinson’s Disease Rating Scale (UPDRS), freezing of gait questionnaire (FOGQ), Tinetti test, and timed up and go test (TUG) (10, 11); (2) spatio-temporal parameters of gait, in particular cadence and gait speed (9, 17); and (3) amplitude and timing of the muscular contraction, in particular with regards gastrocnemius, tibialis anterior, and vastus lateralis (2, 12). Recently, improvements have been observed also in terms of kinematics. Indeed, it has been shown that the typical gait of PD patients (33) would be modified by a rehabilitation program integrated with RAS, with the hip flexion–extension movement closer to the normality after rehabilitation (13).

Overall, the positive effects of RAS training based on metronome or music (artificial RAS) are quite well-established in the PD literature. Given that the tempo of RAS is usually determined on the basis of patient’s own cadence, in a certain degree RAS represents the perceptualization of biological information associated with gait. However, from the perceptual point of view, the experience of artificial sounds like metronome or music is quite far from the auditory experience of walking. Indeed, artificial RAS only provide rhythmic information, while more ecological stimuli such as footstep sounds would provide both rhythmic information and other gait-related information (e.g., posture, force, gait cycle). Surprisingly, to the best of our knowledge, nobody has explored whether RAS based on ecological footstep sounds can be advantageous, compared to RAS based on artificial sounds (e.g., metronome), within a PD rehabilitation program.

The effects of ecological versus artificial sounds on motor processes have been explored in various domains, such as breathing (34) and motor learning (35), with apparently contradictory results probably because of the different methods employed. In the domain of PD, the role of ecological sounds has been explored in laboratory experiments on walking, revealing interesting results. In one of these experiments, Rodger, Young, and Craig (36) found that the use of synthesized footstep sounds reduced the coefficient of variation of stride length and stride duration, compared to normal walk without sounds. In another study on PD patients (37), the same authors found that the variability of step length and step duration was lower when administering footstep sounds compared to metronome sounds, in a real-time imitation task. This evidence suggests that the complexity of ecological sounds can provide more information than simple beats and this pattern of information can be used as guidance for walking. Recently, it has been argued that the information conveyed by footstep sounds may have important implications for the enhancement of gait in PD (4, 38), and it has been questioned whether a rehabilitation program based on ecological sounds may be more advantageous compared to a metronome-based program.

The rationale of the hypothesized greater effects of the ecological sounds originates from both neurophysiological evidence and perceptual-motor theories. As regards the neurophysiological evidence, it is well-known that the neurons with mirror properties (39) are associated with imitation (40). Indeed, the main areas of the mirror system (i.e., inferior parietal lobule, precentral gyrus, inferior frontal gyrus) activate when humans see or perform an action, but even when they hear the same action (41–44). Based on this evidence, it seems reasonable to hypothesize that listening to footstep sounds would activate some of the brain regions involved in the control of walking, possibly triggering imitative behaviors.

As regards the perceptual-motor theories, it has been suggested that the perceptual and the motor systems share a common representational organization, and continuously influence each other (45, 46). Thus, the footstep sounds would evoke a representation of walking in the common representational system, which would reinforce a representation of the same gesture already stored in memory, due to previous perceptual-motor experience. The same representation would not be triggered by the metronome sounds, because they are not intrinsically related to walk and would not be able to adequately resemble a human walking representation. As a consequence, footstep sounds would activate a powerful representation of human walking in the common representational system (45, 46), which with a higher probability would influence the corresponding motor outcomes (4, 34), namely, walking. In sum, while the artificial RAS (i.e., metronome) would provide patients with rhythmic cues only, the ecological RAS (i.e., footsteps) would provide patients with rhythmic cues, which are also meaningful and able to evoke a mental representation of walking.

In this study, we aim to investigate whether the hypothesized superiority of the ecological over artificial RAS has an actual impact in a PD rehabilitation program. In particular, we intend to better understand whether the type of sound (i.e., footsteps or metronome) is relevant in a typical rehabilitation protocol including a gait training with RAS. We hypothesize that patients treated with footstep sounds would improve more than those treated with metronome sounds. Indeed, we expect that both groups would benefit from the rhythmic information of the stimuli, but the former would also take advantage of the priming effect elicited by the ecological information of footstep sounds.

## Materials and Methods

### Participants

**Figure 1:**
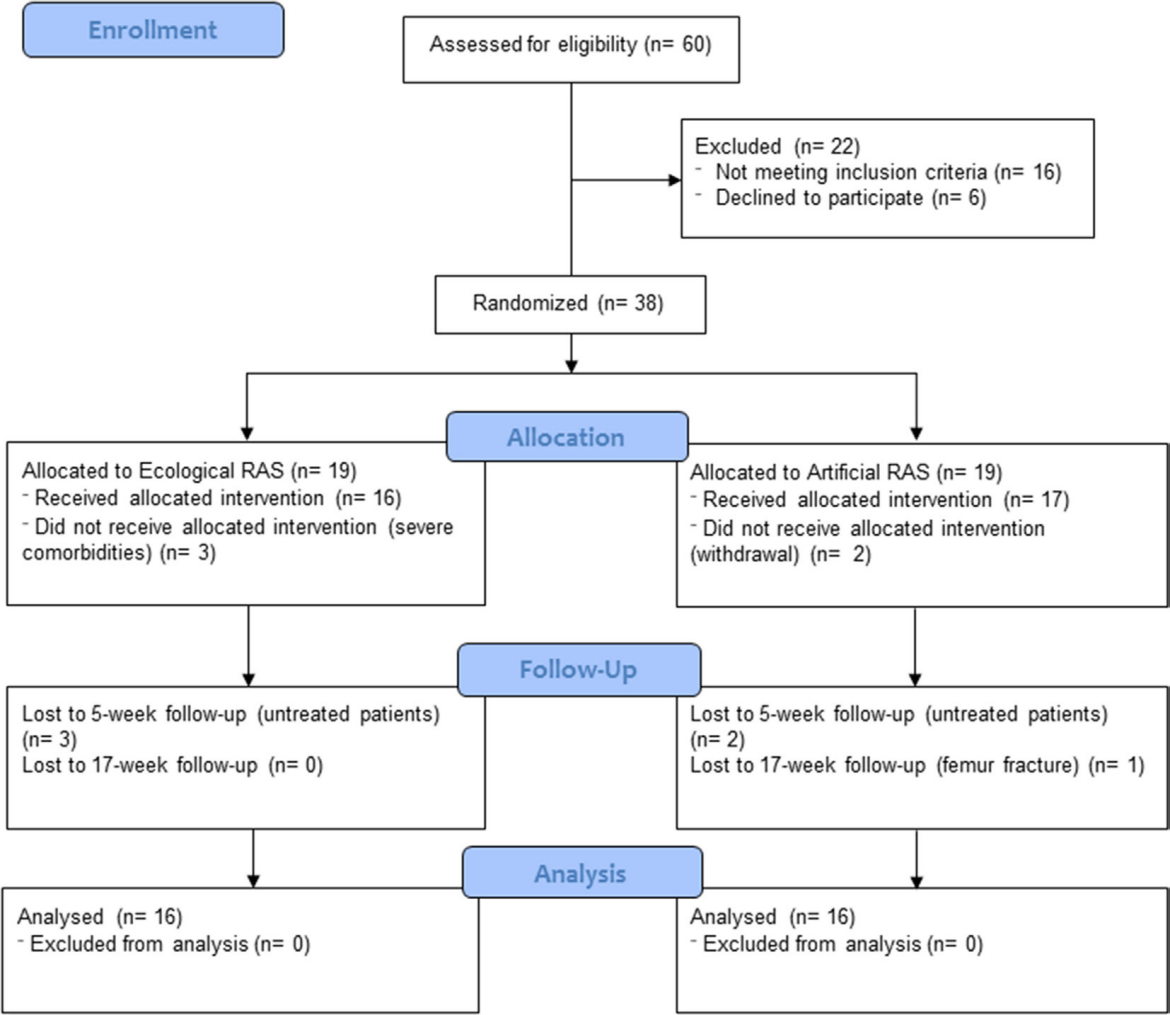
Participant flow diagram.

Thirty-eight individuals affected by PD participated in this study and 32 of them (*M*_age_ = 68.2 years; SD = 10.5) completed it; see Figure 1 for participant flow diagram and Table 1 for baseline demographic and clinical characteristics of participants. Patients were enrolled by RP, CC, and GC between December 2014 and February 2015 at “G. Brotzu” General Hospital (Cagliari, Italy), where they were informed about the present study. The sample size was calculated by means of the G*Power software (parameters: alfa = 0.05; power = 0.80; effect size = 0.25), and the result was 28 participants. All patients included in the study met the following criteria: diagnosis of PD according to the UK Brain Bank criteria (47) ability to walk independently; absence of relevant hearing impairments which could prevent the correct perception of the auditory cues (i.e., ability to have a regular conversation with medical doctors during interview without the use of hearing aids and without the doctor shouting); absence of significant cognitive impairment [i.e., mini-mental status examination (MMSE) >24; frontal assessment battery (FAB) >13]; absence of psychiatric or severe systemic illnesses; mild-to-moderate disability assessed by means of the modified Hoehn and Yahr (H&Y) staging scale (1.5 ≤ H&Y ≤ 3); and no engagement in any rehabilitative program in the 3 months before the beginning of the study. When participants were recruited, all of them were treated with l-DOPA and five of them were also taking dopamine agonists. The experimental protocol was approved by local ethics committee (Prot. PG/2014/19654). Written informed consent was obtained by all participants.

**Table 1:** Baseline demographic and clinical characteristics for each group.

	Ecological rhythmic auditory stimulation (RAS) group	Artificial RAS group
Age (years)	66.5 ± 10.9	69.9 ± 10.1
Parkinson’s disease (PD) duration (years)	6.9 ± 5.4	5.8 ± 6.2
Hoehn & Yahr (H&Y)	1.5 ≤ H&Y ≤ 2.5	1.5 ≤ H&Y ≤ 3
Unified PD rating scale (UPDRS III)	18.0 ± 9.1	20.2 ± 9.6
Mini-mental status examination	27.1 ± 1.6	27.9 ± 1.5
Frontal assessment battery	17.1 ± 1.5	16.5 ± 1.2

### Stimuli

#### Footsteps Recording

The recordings were carried out in a soundproof room with a parquet floor. Footstep sounds were recorded by means of a fixed-cardioid, large-diaphragm condenser sE2200A microphone. The microphone was fixed on an elastic shock mount in order to isolate it from mechanically transmitted noise; the elastic shock, in turn, was fixed on a stick. The microphone was connected to a M-AUDIO Fast Track Ultra 8R external sound card, which was connected to a laptop running the Logic Pro X software.

A database of footstep sounds was created using the following procedure. Fourteen healthy young adults (7F, 7M) participated in the recording phase. These volunteers were recruited on the basis of their weight. Specifically, females’ weight ranged from 45 to 75 kg with intervals of 5 kg, while males’ weight ranged from 60 to 90 kg, always with 5 kg intervals. The volunteers were required to wear garments free of synthetic fabrics (to avoid potential side noises) and a pair of their own sneakers with a rubber sole. Each volunteer was required to take six steps of 70 cm at the pace of 100 BPM. A set of strips were marked on the floor to provide the correct distance, while the pace was provided by means of earplugs conveying a metronome sound from a portable MP3 player. The recordings were carried out by an experimenter following the walk of the volunteer from a side, without walking, by using the microphone stick.

#### Stimuli Editing

**Figure 2:**
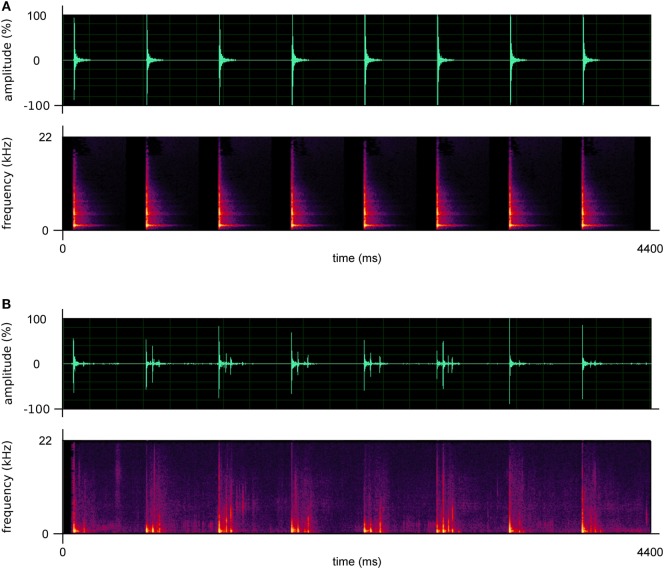
Prototypical examples of the artificial **(A)** and ecological sounds **(B)** used in this study. The upper graphs describe the amplitude of the sounds; the lower graphs describe their frequencies.

Two kinds of RAS stimuli (i.e., ecological and artificial) were created. Ecological stimuli consisted of footstep recordings taken from the above-described database; artificial stimuli consisted of metronome sounds. Each patient was provided with one stimulus, either ecological or artificial (depending on the assigned condition); the stimuli were personalized for each patient. In this regard, ecological stimuli were assigned to patients on the basis of their own gender and weight, thus providing patients with sounds similar to those produced by themselves. Moreover, for both ecological and artificial stimuli, the BPM of the soundtracks provided to patients were calculated considering one’s own cadence measured before the beginning of the rehabilitation program (at T0, see “experimental design” paragraph), and the cadence of healthy individuals of the same age (48, 49). In particular, the BPM were calculated following the procedure of Pau and colleagues (13), namely: (a) if a patient’s cadence was below the normality, the BPM of the stimulus was set at a value of 10% higher than one’s own cadence; (b) if a patient’s cadence was below, but close to normality (less than 10% difference), the BPM of the stimulus was set at normality values; (c) if a patient’s cadence was above the normality, the BPM of the stimulus was set at a value equal to one’s own cadence. The interval between one beat/step and the subsequent one was constant, in both conditions. Prototypical examples of artificial and ecological sounds are illustrated in Figure 2 and attached in Supplementary Material.

### Assessment Protocol

Assessment was carried out when patients were in “ON” state 60–90 min after intake of the usual morning l-DOPA dose. The assessment was carried out at the Laboratory of Biomechanics and Industrial Ergonomics of the Department of Mechanical, Chemical and Materials Engineering, University of Cagliari (Italy), lasted about 90–100 min and included both biomechanical and clinical evaluations.

#### Biomechanical Evaluation

We measured the spatio-temporal parameters of gait. The acquisition of these parameters was performed using a motion capture system composed of 8 infrared cameras (Smart-D system, BTS Bioengineering, Italy) set at a frequency of 120 Hz. Before the tests, a number of anthropometric features (i.e., height, weight, anterior superior iliac spines distance, pelvis thickness, knee and ankle width, leg length) were collected. Then, 22 reflective passive markers (14 mm diameter) were placed on specific landmarks of individual’s lower limbs and trunk according to the protocol described by Davis et al. (50). Participants were asked to walk at a self-selected speed in the most natural manner possible on a 10 m walkway for at least six times, allowing suitable rest were needed in order to avoid fatiguing effects. The raw data were then processed using dedicated software (Smart Analyzer, BTS Bioengineering, Italy) to calculate the following spatio-temporal parameters, which were the primary outcome measures of the present study: gait speed, step length and width, stride length, cadence, stance, swing, and double support phase duration (the latter three parameters expressed as percentage of the gait cycle duration).

#### Clinical Evaluation

A clinical evaluation of the patients was performed by a team of clinicians’ expert in PD. Clinical measures were the secondary outcome measures of the present study. The patients were evaluated by using the following tests:

- Modified H&Y to assess the severity of symptoms of each patient on one of the seven levels of the scale (51, 52).
- Unified Parkinson’s disease rating scale (UPDRS) part 3 to specifically evaluate the motor symptoms (53).
- Functional independence measure (FIM) to measure the patients’ disability, revealed by their independence, by the amount of care needed and by their cognitive skills (54).
- Tinetti test to evaluate the static and dynamic balance (55).
- Short physical performance battery (SPPB) to evaluate the functionality of the lower limbs. This test is composed of three sections: balance (assessed in three different trials), walking speed (4 m test), and sit to stand (STS) transfer (56).
- Geriatric depression scale (GDS)—short version (15 items), a self-report scale to assess depression in elderly (57).
- Parkinson’s disease quality of life questionnaire (PDQ-8), a specific scale to evaluate the quality of life of patients affected by PD (58).
- Falls efficacy scale (FES) to evaluate the patients’ fear of falling (59).
- Activities specific balance confidence (ABC), a self-efficacy scale regarding activities which require specific balance confidence (60).
- Freezing of gait questionnaire (FOGQ) to evaluate freezing episodes reported by patients during walking (61).

### Rehabilitation Protocol

Participants were engaged in a supervised rehabilitative treatment which lasted 5 weeks; during this period patients were engaged in two sessions per week, whose duration was 45 min each. Patients were individually assisted in the training by a physical therapist, under the supervision of a physical medicine specialist; the treatment sessions were held at “G. Brotzu” General Hospital.

The treatment sessions consisted of standard and personalized exercises aimed at enhancing mobility, balance, and posture. Twenty minutes of each session were dedicated to specific gait training with RAS, with participants engaged in walking while listening to their own personalized soundtrack (either ecological or artificial). Moreover, during the 5 weeks of treatment, participants were invited to train at least three times a week at their homes, performing a subset of the same exercises typically performed at the hospital and 30 min of gait training with RAS (they were provided with an MP3 player). Participants were asked to set the volume at a comfortable level and were allowed to modify it anytime.^1^ The rehabilitation protocol was similar to that used by Pau and colleagues (13); a detailed description of the exercises is reported in Appendix.

After the 5 weeks in which participants were engaged in the supervised treatment, they were invited to daily perform their home-exercises for the subsequent 12 weeks. The activities performed by patients during these 12 weeks were unsupervised, however, participants were asked to keep a log of their home-exercises and such data were discussed with clinicians during regularly scheduled meetings.

### Experimental Design

Participants were randomly assigned to the groups (ecological or artificial RAS) in a 1:1 fashion, using blocked randomization (62). Randomization was generated by MM, by means of an online sequence generator (www.random.org) inserting 1 and 38 as smallest and largest numbers and calculating random sequences in 2 columns of 19 numbers each. MM also assigned participants to interventions. Participants were evaluated in three moments: before the rehabilitative treatment (T0), at the end of the 5-week rehabilitative treatment (T5), and 3 months after the end of the treatment, namely 17 weeks after the first assessment (T17). Thus, in this study there were two independent variables: (1) RAS, between subjects, two levels (ecological RAS, artificial RAS); (2) Time, within subjects, three levels (T0, T5, T17). The dependent variables were all the biomechanical and clinical measurements described above. The researcher who assigned participants to the conditions was not involved in the enrollment and evaluation of patients, while the researchers involved in the evaluation of patients (MP, FC, RP, CC, GC) were not aware of the conditions under which the patients were treated (observer-blind trial). Data collection was concluded in April 2016.

### Statistical Analyses

As regards the biomechanical variables, a preliminary *t*-test was run to test for possible differences between the left and right limbs (when separate data were available). As no significant difference was revealed by the analyses, we used the mean of the two limbs for each parameter, for each participant, in the subsequent analyses. We conducted a 3 × 2 mixed MANOVA, using all biomechanical variables as dependent measures. *Post hoc* comparisons were adjusted with LSD test; the alpha level was set at 0.05.

As regards the clinical variables, for each dependent variable, a 3 × 2 mixed ANOVA (Time × RAS) was applied. *Post hoc* analyses were calculated by using repeated measures ANOVAs and *t*-tests. The alpha level was set at 0.05 for the omnibus tests and was adjusted with the Bonferroni formula for *post hoc* analyses (*p* value = 0.05/*n* comparisons).

Moreover, independently of the outcomes of the previous analyses, we planned to conduct a set of additional exploratory analyses, to better examine the potential of the footstep sounds. To this purpose, we separately tested the two groups of participants, by conducting two repeated measures MANOVAs and a set of contrasts adjusted with LSD test on the primary outcome measures of this study (i.e., the biomechanical measures).

All the analyses were performed using the SPSS Statistics software.

## Results

A preliminary set of analyses was run to compare the two groups at baseline for each variable, and no significant difference was found. The effects of the two treatments (rehabilitation with ecological vs. artificial RAS) across time (T0, T5, T17) were observed using both biomechanical and clinical measures.

### Biomechanical Measures

Overall, the analyses run on biomechanical measures (Table 2) did not reveal any significant result for the interaction Time x RAS and for the main effect of RAS. Although the results did not reveal a statistical significance for interaction—which was the primary interest of our investigation—the majority of the considered parameters indicated a significant effect of the variable Time [Wilk’s λ = 0.098, *F*(16, 15) = 8.652, *p* < 0.001, $\eta_{p}^{2}=0.\text{9}0\text{2}$]. In particular, this was found for cadence [*F*(2, 60) = 4.595; *p* = 0.01; $\eta_{p}^{2}=0.\text{133}$], gait speed [*F*(2, 60) = 8.538; *p* < 0.001; $\eta_{p}^{2}=0.\text{222}$], step width [*F*(2, 60) = 12.647; *p* < 0.001; $\eta_{p}^{2}=0.\text{297}$], step length [*F*(2, 60) = 17.752; *p* < 0.001; $\eta_{p}^{2}=0.\text{372}$], stride length [*F*(2, 60) = 3.681; *p* < 0.05; $\eta_{p}^{2}=0.\text{1}0\text{9}$], percentage of double support phase [*F*(2, 60) = 4.911; *p* = 0.01; $\eta_{p}^{2}=0.\text{141}$], and percentage of swing phase [*F*(2, 60) = 6.843; *p* < 0.005; $\eta_{p}^{2}=0.\text{186}$]. Conversely, the effect of time on the percentage of stance phase was not significant.

**Table 2:** Comparison between spatio-temporal parameters assessed before and after rehabilitation for each group.

	Spatio-temporal parameters of gait			
		T0	T5	T17
Ecological RAS group	Gait speed (m/s)	1.08 ± 0.24	1.21 ± 0.25	1.24 ± 0.22
	Cadence (steps/min)	115.81 ± 11.71	123.40 ± 9.07	123.49 ± 11.98
	Stride length (m)	1.16 ± 0.20	1.20 ± 0.19	1.21 ± 0.17
	Step length (m)	0.52 ± 0.12	0.58 ± 0.10	0.60 ± 0.09
	Step width (m)	0.18 ± 0.03	0.18 ± 0.04	0.20 ± 0.02
	Stance phase (% of gait cycle)	60.48 ± 3.31	59.59 ± 1.74	59.98 ± 1.87
	Swing phase (% of gait cycle)	38.68 ± 2.59	40.42 ± 1.74	39.98 ± 1.90
	Double support (% of gait cycle)	11.34 ± 2.44	10.07 ± 2.44	9.96 ± 2.02

Artificial RAS group	Gait speed (m/s)	1.05 ± 0.29	1.11 ± 0.24	1.15 ± 0.29
	Cadence (steps/min)	113.43 ± 14.54	115.53 ± 11.34	116.50 ± 11.62
	Stride length (m)	1.13 ± 0.20	1.16 ± 0.18	1.18 ± 0.21
	Step length (m)	0.52 ± 0.12	0.56 ± 0.10	0.59 ± 0.11
	Step width (m)	0.17 ± 0.03	0.17 ± 0.03	0.20 ± 0.02
	Stance phase (% of gait cycle)	60.79 ± 2.92	60.32 ± 2.36	60.12 ± 2.20
	Swing phase (% of gait cycle)	38.89 ± 2.57	39.63 ± 2.33	39.43 ± 2.62
	Double support (% of gait cycle)	11.73 ± 2.78	10.69 ± 2.42	10.70 ± 2.55

*Values are expressed as mean ± SD*.

Then, we investigated more deeply how these parameters changed across time. We found that the cadence significantly increased between T0 and T5 (*p* = 0.021) and between T0 and T17 (*p* = 0.029), while it remained constant between T5 and T17. Analogous results were revealed by the analysis on gait speed, indeed it significantly increased between T0 and T5 (*p* = 0.006) and between T0 and T17 (*p* = 0.001) and remained constant between T5 and T17. Similarly, the percentage of swing phase significantly increased between T0 and T5 (*p* = 0.001) and between T0 and T17 (*p* = 0.033), while it remained constant between T5 and T17. Consistently, the percentage of double support phase significantly decreased between T0 and T5 (*p* = 0.009) and between T0 and T17 (*p* = 0.013) and remained constant between T5 and T17. As concerns step length, we found an increase between T0 and T5 (*p* = 0.004), between T0 and T17 (*p* < 0.001), and also between T5 and T17 (*p* = 0.01). Surprisingly, we did not find a difference between T0 and T5 regarding step width (*p* = 0.568), but the values observed in T17 were significantly higher than those observed in T0 (*p* < 0.001) and T5 (*p* < 0.001). Moreover, as concerns stride length, there was no difference between T0 and T5 (*p* = 0.108), while it significantly increased between T0 and T17 (*p* = 0.011); no difference was found between T5 and T17.

### Clinical Measures

The analyses run on clinical measures (Table 3) did not reveal any significant result for the time × RAS interaction and for the main effect of RAS. As regards time, similarly to the analyses on biomechanical measures, we found that the main effect was significant for almost all clinical measures. In particular, a significant main effect of time was observed for the UPDRS—part 3 [*F*(2, 52) = 32.749; *p* < 0.001; $\eta_{p}^{2}=0.\text{557}$], ABC [*F*(2, 56) = 5.418; *p* < 0.01; $\eta_{p}^{2}=0.\text{162}$], FES [*F*(2, 58) = 4.819; *p* < 0.05; $\eta_{p}^{2}=0.\text{143}$], FOGQ [*F*(2, 60) = 3.926; *p* < 0.05; $\eta_{p}^{2}=0.\text{116}$], GDS [*F*(2, 58) = 3.663; *p* < 0.05; $\eta_{p}^{2}=0.\text{112}$], PDQ8 [*F*(2, 58) = 7.343; *p* < 0.001; $\eta_{p}^{2}=0.\text{2}0\text{2}$], Tinetti test [*F*(2, 58) = 3.945; *p* < 0.05; $\eta_{p}^{2}=0.\text{12}$], SPPB [*F*(2, 58) = 5.330; *p* < 0.01; $\eta_{p}^{2}=0.\text{155}$] and its subcomponents STS [*F*(2, 42) = 15.390; *p* < 0.001; $\eta_{p}^{2}=0.\text{423}$], and 4-m test [*F*(2, 40) = 7.382; *p* < 0.01; $\eta_{p}^{2}=0.\text{27}0$]. Conversely, no significant effect was found for the FIM scale.

**Table 3:** Comparison between clinical scores assessed before and after rehabilitation for each group.

	Clinical scores			
		T0	T5	T17
Ecological rhythmic auditory stimulation (RAS) group	UPDRS—part 3	16.93 ± 9.14	11.64 ± 7.00	10.54 ± 6.96
	Functional independence measure (FIM)	123.47 ± 3.91	123.87 ± 3.27	123.87 ± 3.25
	Tinetti test	27.19 ± 1.76	27.75 ± 0.45	27.62 ± 0.62
	Short physical performance battery (SPPB)	11.33 ± 1.54	11.87 ± 0.35	11.87 ± 0.35
	SPPB—4 m test (s)	2.56 ± 0.28	2.25 ± 0.22	2.42 ± 0.22
	SPPB— sit to stand (STS) (s)	9.83 ± 1.80	8.47 ± 1.20	8.17 ± 1.34
	Geriatric depression scale (GDS)	3.33 ± 2.26	3.33 ± 2.53	2.47 ± 2.20
	Parkinson’s disease quality of life questionnaire (PDQ)-8	20.21 ± 13.46	20.00 ± 11.92	14.17 ± 11.56
	Falls efficacy scale (FES)	4.73 ± 6.95	2.40 ± 3.22	2.27 ± 2.34
	Activities specific balance confidence (ABC)	81.96 ± 13.21	85.23 ± 7.49	88.38 ± 8.75
	Freezing of gait questionnaire (FOGQ)	4.00 ± 5.14	2.44 ± 3.76	2.62 ± 4.08

Artificial RAS group	UPDRS—part 3	20.21 ± 9.59	15.36 ± 9.20	12.79 ± 6.78
	FIM	117.94 ± 13.12	118.50 ± 10.91	119.00 ± 10.48
	Tinetti test	26.93 ± 1.58	27.47 ± 0.74	27.67 ± 0.49
	SPPB	10.94 ± 2.29	11.75 ± 0.77	11.69 ± 0.70
	SPPB—4 m test (s)	2.72 ± 0.70	2.30 ± 0.36	2.34 ± 0.44
	SPPB—STS (s)	10.00 ± 1.52	8.43 ± 1.21	8.47 ± 1.42
	GDS	3.56 ± 2.85	2.75 ± 2.27	2.19 ± 2.23
	PDQ-8	17.97 ± 10.86	16.21 ± 11.87	12.69 ± 10.67
	FES	5.50 ± 5.49	3.62 ± 3.01	3.56 ± 3.60
	ABC	79.37 ± 14.98	83.81 ± 8.73	87.12 ± 8.37
	FOGQ	3.00 ± 4.27	2.56 ± 3.72	2.81 ± 4.00

*Values are expressed as mean ± SD*.

On the basis of the ANOVAs results, we decided to further explore how the clinical measures changed across time. We found that the FES scores decreased between T0 and T5 [*t*(30) = 2.375; *p* < 0.05; *d* = 0.377] and between T0 and T17 [*t*(30) = 2.367; *p* < 0.05; *d* = 0.406], while they remained constant between T5 and T17. Analogous results were observed for SPPB, with an increase between T0 and T5 [*t*(30) = 2.456; *p* = 0.01; *d* = 0.304] and between T0 and T17 [*t*(30) = 2.367; *p* < 0.05; *d* = 0.253], and no difference between T5 and T17. This trend was also observed in two of the subcomponents of SPPB, namely STS and 4-m test. There was a decrease of time necessary to complete the test between T0 and T5 for both the STS [*t*(22) = 4.082; *p* < 0.001; *d* = 1.016] and the 4-m test [*t*(22) = 2.331; *p* < 0.05; *d* = 0.647], and between T0 and T17 for both the STS [*t*(22) = 4.651; *p* < 0.001; *d* = 1.061] and the 4-m test [*t*(22) = 2.666; *p* < 0.01; *d* = 0.569]; in no case a difference between T5 and T17 was observed.

The FOGQ scores also decreased between T0 and T5 [*t*(31) = 2.459; *p* = 0.01; *d* = 0.217], while the difference between T0 and T17 was no longer significant after the Bonferroni correction [*t*(31) = 1.797; *p* < 0.05; *d* = 0.173]; no difference was observed between T5 and T17. A similar trend was observed for the Tinetti test, with marginally significant increases between T0 and T5 [*t*(30) = 2.129; *p* < 0.05; *d* = 0.376] and between T0 and T17 [*t*(30) = 2.037; *p* < 0.05; *d* = 0.438], which were no longer significant after the Bonferroni adjustment; again no difference was observed between T5 and T17.

The UPDRS—part 3 scores decreased between T0 and T5 [*t*(27) = 7.701; *p* < 0.001; *d* = 0.548], between T0 and T17 [*t*(27) = 6.261; *p* < 0.001; *d* = 0.781], and also between T5 and T17 [*t*(31) = 2.598; *p* < 0.01; *d* = 0.269]. Analogously, the ABC scores increased between T0 and T5 [*t*(29) = 1.997; *p* < 0.05; *d* = 0.302], between T0 and T17 [*t*(29) = 2.556; *p* < 0.01; *d* = 0.609] and between T5 and T17 [*t*(30) = 2.240; *p* < 0.05; *d* = 0.385], however, the first and the last comparisons were no longer significant after Bonferroni correction.

The PDQ8 revealed the best results at T17, with higher scores compared to both T0 [*t*(30) = 2.950; *p* < 0.01; *d* = 0.490] and T5 [*t*(31) = 2.966; *p* < 0.01; *d* = 0.285], while no difference was observed between T0 and T5. As concerns the GDS, a marginal decrease was observed between T0 and T17 [*t*(30) = 2.171; *p* < 0.05; *d* = 0.476], which disappeared after the Bonferroni correction, while the other comparisons did not reach any significant value.

### Additional Exploratory Analyses

**Figure 3:**
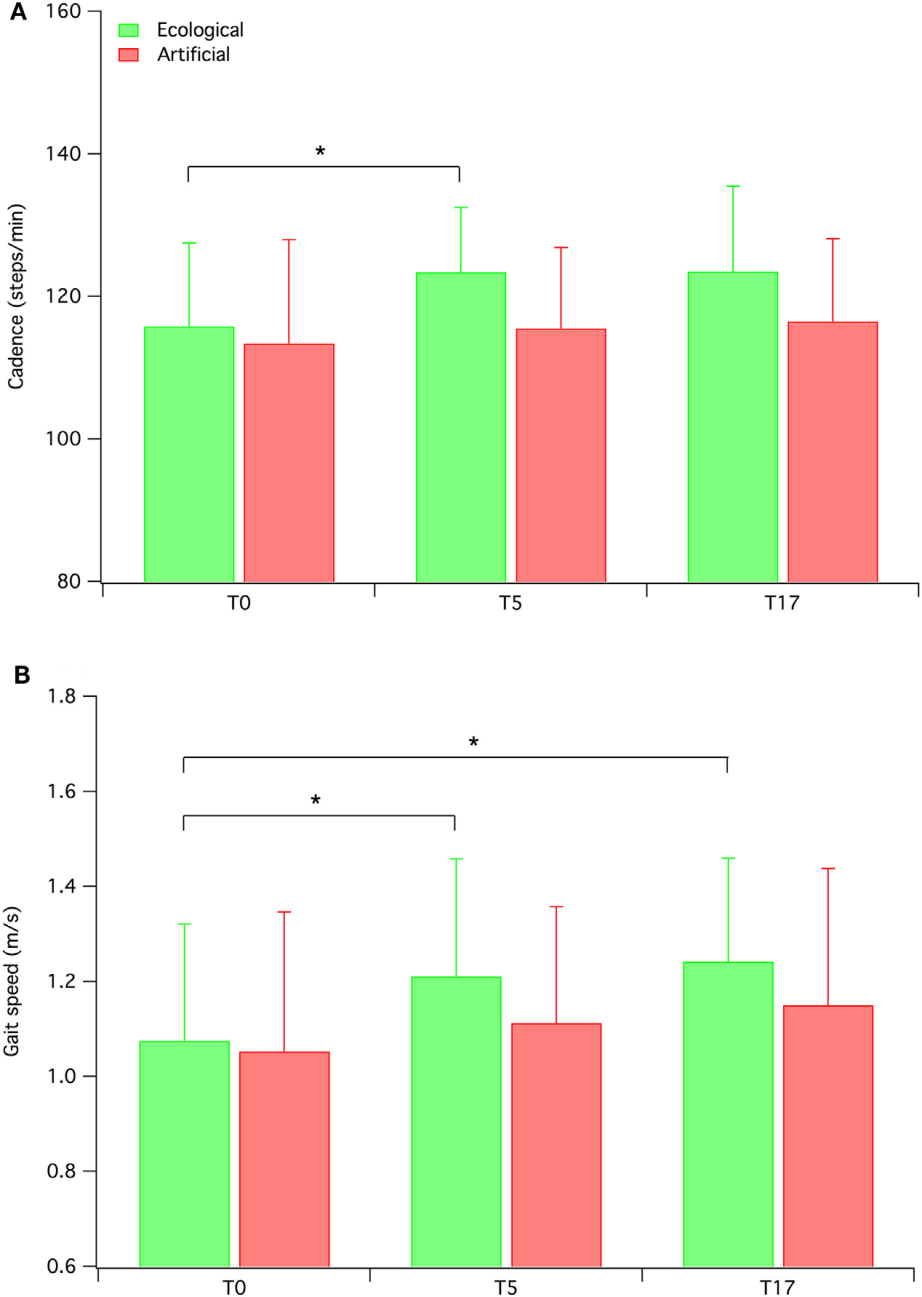
Mean cadence **(A)** and gait speed **(B)** for both ecological rhythmic auditory stimulation (RAS) participants and artificial RAS participants. Error bars represent SD.

We conducted a set of exploratory analyses by separately examining the two groups of participants on the primary outcome measures (i.e., biomechanical measures). The repeated measures MANOVAs revealed significant results for the ecological RAS group [Wilk’s λ = 0.23, *F*(16, 46) = 3.149, *p* = 0.001, $\eta_{p}^{2}=0.\text{523}$], but not for the artificial RAS group. The MANOVA conducted on the ecological RAS group data revealed significant values for the following measures: cadence [*F*(2, 30) = 4.367; *p* < 0.05; $\eta_{p}^{2}=0.\text{225}$], gait speed [*F*(2, 30) = 5.914; *p* < 0.01; $\eta_{p}^{2}=0.\text{283}$], percentage of swing phase [*F*(2, 30) = 5.533; *p* < 0.01; $\eta_{p}^{2}=0.\text{269}$], step length [*F*(2, 30) = 10.23; *p* < 0.001; $\eta_{p}^{2}=0.\text{4}0\text{5}$], and step width [*F*(2, 30) = 5.322; *p* < 0.05; $\eta_{p}^{2}=0.\text{262}$]. In our opinion, of particular interest are the results concerning cadence and gait speed (see Figure 3), since these are the two measures more directly related to the auditory stimuli. The contrasts for these variables indicate that cadence significantly increased from T0 to T5 (*p* = 0.011), but this advantage was not maintained at T17 (*p* = 0.072), while gait speed increased from T0 to T5 (*p* = 0.017) and this advantage was maintained at T17 (*p* = 0.13).

## Discussion

The aim of this study was to investigate whether a PD rehabilitation program integrated with ecological RAS (i.e., footstep sounds) can be more effective than the same program integrated with artificial RAS (i.e., metronome sounds). We hypothesized that both groups would benefit from the rhythmic information of the stimuli, but the group exposed to ecological RAS would also take advantage of the priming effect elicited by the ecological information of footstep sounds. The results observed on the primary outcome measures (biomechanical measures) suggest that the treatments are equally effective as no significant interaction was revealed by analyses. The same results were given by the secondary outcome measures (clinical measures).

As a whole, our results indicate that—independently of the type of sound— the rehabilitation programs integrated with RAS are effective. Indeed, comparing the biomechanical and clinical data collected before and after the treatment, we observed noticeable improvements in the majority of the variables. This evidence is in line with previous literature, which clearly proved the efficacy of rehabilitation with RAS, in terms of both spatio-temporal parameters and clinical variables (2, 9–11, 17). Moreover, we observed that these improvements were largely maintained at the follow-up, 3 months after the end of the supervised period, which represents a longer term compared to the majority of previous RAS studies. However, given that the RAS efficacy is well-documented in literature, our main aim was not to further confirm it, but to examine whether the type of sound (i.e., ecological or artificial) can actually influence the efficacy of rehabilitation.

The novelty of this study is that, for the first time, we used biological motion sounds as RAS in a rehabilitation program with PD patients. The same rehabilitation program was integrated either with footstep sounds (ecological RAS condition) or with metronome sounds (artificial RAS condition) and the overall results indicated that the effects of the two sounds—in a rehabilitation context—are equivalent. However, to better examine the potential of the footstep sounds and obtain additional information that could be used as starting point for future investigations, we ran a set of exploratory analyses on biomechanical measures for each group, separately. These analyses revealed that only the patients assigned to the ecological RAS condition significantly improved. Among the various measures showing significant improvements, in our opinion a particular attention should be dedicated to cadence and gait speed, since these are the two parameters more directly linked to the auditory stimuli. However, we acknowledge that our exploratory analyses are only informative but not conclusive; indeed these analyses do not compare the two groups and cannot prove a superior effect of ecological sounds over artificial sounds. Future studies should better clarify the potential of footstep sounds in the rehabilitation context.

The effects of ecological and artificial sounds on motor tasks in PD patients were examined in previous research (37). In their study, Young and colleagues found that in some spatio-temporal parameters patients performed better in the ecological sound condition than in the artificial sound condition. However, the methods used in the previous and in this study are quite different: the patients tested by Young and colleagues were engaged in a real-time imitation task, while in our case the patients were engaged in a 5-week rehabilitation program. To the best of our knowledge this is the first time that a rehabilitation program is integrated with ecological RAS, since previous RAS experiments generally used music or metronome sounds (4–6). Therefore, our study can be considered as a first attempt to investigate in this direction and further research is needed to understand whether this line of investigation can be fruitful.

Our hypothesis, based on the audiovisual mirror neurons (41–44) and on the common coding perceptual-motor theories (45, 46), was that the use of ecological RAS would constitute an advantage. In particular, we hypothesized that footstep sounds would evoke a mental representation of walking, directly activating the motor systems and, consequently, facilitating patients’ walking. The overall results we observed are not consistent with this hypothesis. Only the additional exploratory analyses are in line with this hypothesis, however, further research stressing the role of RAS is necessary to confirm our exploratory observations. Indeed, we examined the effects of ecological RAS integrated with a standard rehabilitation protocol performed at a hospital. On the one hand, this has a strong ecological validity, since it represents a typical context in which RAS can be employed; on the other hand, the effects of RAS can be somehow masked by the effects of the other exercises performed by the patients. Thus, in our opinion, future studies should isolate the effects of gait training with RAS from the effects of other exercises, making more salient the possible differences between ecological and artificial RAS.

Like every study, also our work does have some limitations. The severity of the disease of our participants was mild–moderate, so we cannot extend our results to patients with more severe impairments. Moreover, by experimentally testing a sample of individuals with a higher level of motor impairment it could be possible to observe stronger effects. This might be helpful to better understand the possible different effects between the two types of sound. Another limitation is that we did not control/manipulate important parameters of sounds (i.e., volume, frequency). In our opinion, specific research of how these features of the stimuli affect gait training should be performed in more controlled laboratory experiments, rather than in ecological settings as in our study. Moreover, the subjective pleasantness of the two types of sound (ecological vs. artificial) should be investigated.

From an applied perspective, our results suggest that ecological RAS is equally effective compared to artificial RAS, therefore, it would be possible to let patients decide what type of sound they prefer for gait training. This is particularly important for their compliance with the treatment, because the administration of sounds which are perceived as annoying by patients might lead to a low adherence to the training. Moreover, it is noteworthy that several participants reported that the footstep sounds were meaningful and reminded them some physical activities they used to do in the past (e.g., military march), thus evoking motor images directly linked with walking. For future research, the implementation of ecological RAS training in the standard rehabilitation protocols at the hospitals could represent an important source of data, allowing researchers to examine the efficacy of ecological RAS on larger samples of participants.

## Ethics Statement

This study was carried out in accordance with the recommendations of the Independent Ethics Committee of the A.O.U. Cagliari with written informed consent from all subjects. All subjects gave written informed consent in accordance with the Declaration of Helsinki. The protocol was approved by the Independent Ethics Committee of the A.O.U. Cagliari.

## Author Contributions

MM, RP, FC, FS, TA, PB, CC, GC, MG, and MP designed the study. CC and RP performed the physical capacity assessment and prepared the rehabilitation protocol. GC performed the neurological evaluations. MP and FC collected and processed the biomechanical data. MM, TA, FS, and PB prepared the stimuli. MM and MG performed the statistical analyses. MM, FS, and MP wrote the manuscript. RP, FC, TA, PB, CC, GC, and MG revised the manuscript.

## Conflict of Interest Statement

The authors declare that the research was conducted in the absence of any commercial or financial relationships that could be construed as a potential conflict of interest.

## Footnotes

^1^It is noteworthy that participants were asked to use the auditory stimulation for 30 min almost every day, thus it was important that they feel comfortable with it. Moreover, they needed to adapt the volume during home-training depending on the environmental noise.

## Appendix: Rehabilitation Protocol

**Table:**

Targets	Exercises
Prevention of inactivity and fear of falling Prevention of falls Improving physical activity levels Recognizing the onset of fluctuations and adopting suitable movement strategies Learning simple motor exercises of increasing difficulty to be self-administered at home	Segmental exercises of active or assisted mobilization (flexion–extension, prono-supination) to increase strength, mobility, and coordination of four limbs Stretching of anterior and posterior muscular kinetic chains Improvement of static balance: standing (uni- and bipedal), sitting, and quadrupedal posture Improvement of dynamic balance: ambulation on paths of increasing levels of difficulty (e.g., turns, obstacles, etc.) Postural changes: from sitting/quadrupedal to standing, from supine/prone to lateral Occupational therapy exercises Gait training with rhythmic auditory stimulation (for about 50% of the duration of each session)
