# Supplementary material for: The Use of Footstep Sounds as Rhythmic Auditory Stimulation for Gait Rehabilitation in Parkinson’s Disease: A Randomized Controlled Trial
Source: Front Neurol. 2018 May 24;9:348. doi: 10.3389/fneur.2018.00348 (PMC5992388; doi:10.3389/fneur.2018.00348)
Supplement: Supplementary file 1 [file data_sheet_1.docx]

**APPENDIX A: REHABILITATION PROTOCOL**

| **Targets** | **Exercises** |
| --- | --- |
| - Prevention of inactivity and fear of falling - Prevention of falls - Improving physical activity levels - Recognizing the onset of fluctuations and adopting suitable movement strategies - Learning simple motor exercises of increasing difficulty to be self-administered at home | - Segmental exercises of active or assisted mobilization (flexion-extension, prono-supination) to increase strength, mobility, and coordination of four limbs - Stretching of anterior and posterior muscular kinetic chains - Improvement of static balance: standing (uni- and bipedal), sitting, quadrupedal posture - Improvement of dynamic balance: ambulation on paths of increasing levels of difficulty (e.g. turns, obstacles etc.) - Postural changes: from sitting/quadrupedal to standing, from supine/prone to lateral - Occupational therapy exercises - Gait training with RAS (for about 50% of the duration of each session) |
